# The class I PI3K/Akt pathway is critical for cancer cell survival in dogs and offers an opportunity for therapeutic intervention

**DOI:** 10.1186/1746-6148-8-73

**Published:** 2012-05-30

**Authors:** Yu-Ting Chen, Karen AL Tan, Lisa Y Pang, David J Argyle

**Affiliations:** 1 Royal (Dick) School of Veterinary Studies and the Roslin Institute, University of Edinburgh, Edinburgh, UK, EH25 9RG

**Keywords:** Canine, Cancer, PI3, AKT, MTOR, Therapeutic, Target

## Abstract

**Background:**

Using novel small-molecular inhibitors, we explored the feasibility of the class I PI3K/Akt/mTORC1 signaling pathway as a therapeutic target in canine oncology either by using pathway inhibitors alone, in combination or combined with conventional chemotherapeutic drugs *in vitro*.

**Results:**

We demonstrate that growth and survival of the cell lines tested are predominantly dependent on class I PI3K/Akt signaling rather than mTORC1 signaling. In addition, the newly developed inhibitors ZSTK474 and KP372-1 which selectively target pan-class I PI3K and Akt, respectively, and Rapamycin which has been well-established as highly specific mTOR inhibitor, decrease viability of canine cancer cell lines. All inhibitors demonstrated inhibition of phosphorylation of pathway members. Annexin V staining demonstrated that KP372-1 is a potent inducer of apoptosis whereas ZSTK474 and Rapamycin are weaker inducers of apoptosis. Simultaneous inhibition of class I PI3K and mTORC1 by ZSTK474 combined with Rapamycin additively or synergistically reduced cell viability whereas responses to the PI3K pathway inhibitors in combination with conventional drug Doxorubicin were cell line-dependent.

**Conclusion:**

This study highlighted the importance of class I PI3K/Akt axis signaling in canine tumour cells and identifies it as a promising therapeutic target.

## Background

The class I phosphatidylinositol 3-kinase (PI3K) signaling pathway comprises a series of serine/threonine kinase cascades that regulate a variety of cellular processes including cell cycle progression, cell survival and migration, and protein synthesis. Recent evidence supports the hypothesis that the dysregulation of class I PI3K signaling promotes tumourigenesis and angiogenesis in various cancer types [1-3].

Class I PI3K is predominantly activated by receptor tyrosine kinases (RTKs) upon receiving growth factor stimulation. The activated RTKs undergo either autophosphorylation of tyrosine (Y) residues at the intracellular domains or phosphorylation of their substrates such as IRS-1, IRS-2 and Gab on Y residues. The phosphorylated Y residues are soon recognized by SH2 domains in p85 regulatory subunit of class I PI3K, recruiting class I PI3K to plasma membrane, triggering activation of PI3K downstream pathways (reviewed in ref. [4,5]). Alternatively, class I PI3Ks can be activated through the interaction between p110 catalytic subunit and Ras following RTK activation [6-8]. The activated class I PI3K can convert phosphatidylinositol-4,5–biphosphate (PIP2) to phosphatidylinositol-3,4,5–triphosphate (PIP3), resulting in the recruitment of Akt to the plasma membrane and allowing phosphatidylinositol 3-dependent kinase 1 (PDK1) to phosphorylate and activate Akt. In contrast, Akt activity can be counteracted by phosphatase and tensin homolog (PTEN) tumour suppressor through conversion of PIP3 back to PIP2 (reviewed in ref. [9]).

The class I PI3K effects cellular functions through its two major downstream effectors Akt and mTOR. Akt can phosphorylate FoxO3a, BAX, BAD, and caspase 9 to antagonize apoptotic activity, [10-13] phosphorylate pro-survival factors such as MDM2 and IKK-α to maintain cell survival, [14,15] phosphorylate mitochondrial hexokinase-II to prevent mitochondria from initiation of apoptosis, [16] phosphorylate GSK3 and cell cycle inhibitors p21^WAF1^ and p27^KIP^ to promote G1/S cell cycle progression, [17-19] phosphorylate tuberous sclerosis complex 2 (TSC2) or PRAS40 to trigger mTOR complex 1 (mTORC1)-mediated protein synthesis, [20,21] and phosphorylate telomerase reverse transcriptase (TERT) to increase cell longevity [22].

**Figure 1:**
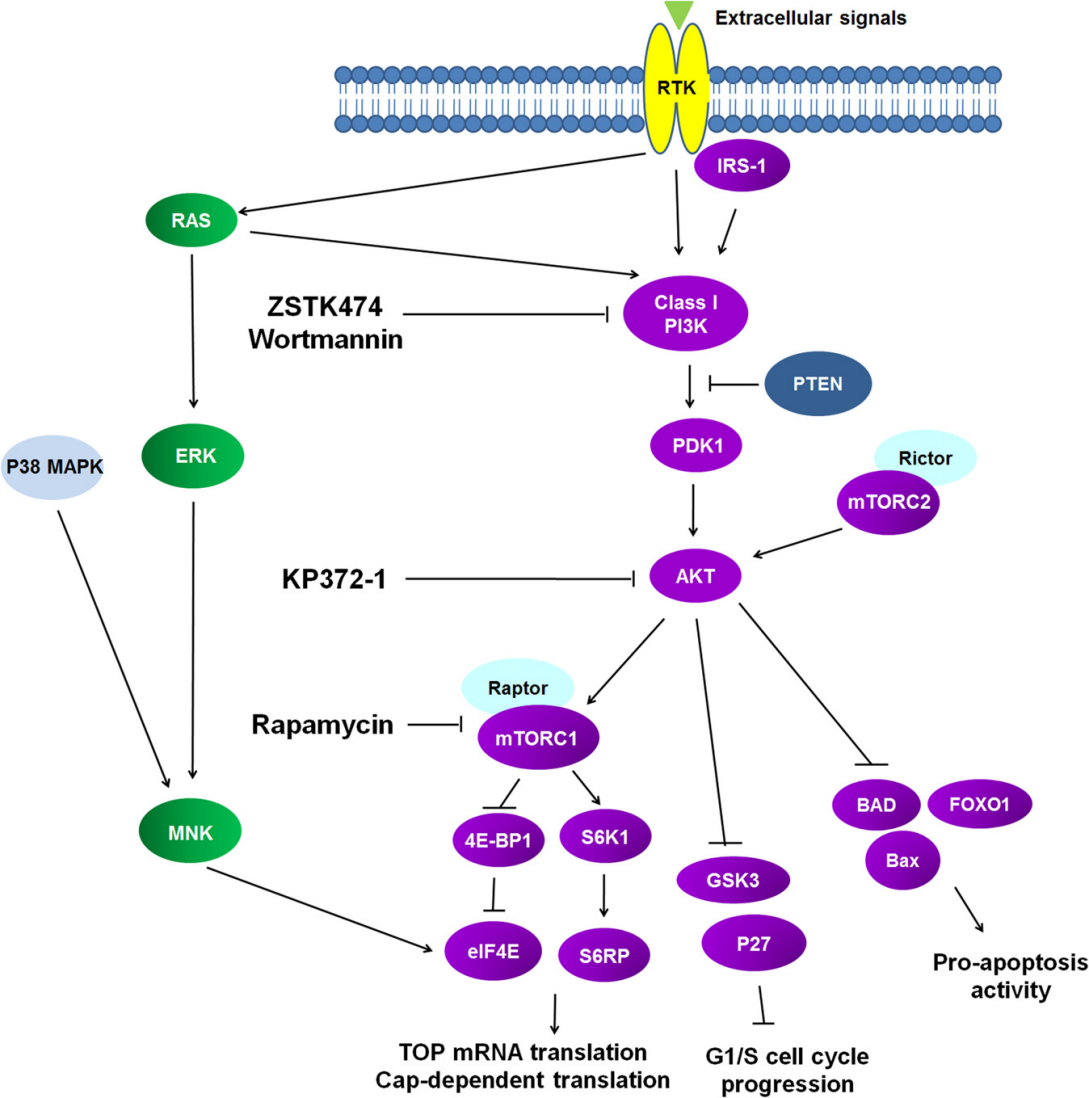
**Schematic diagram of the class I PI3K/Akt/mTOR axis pathway.** The targets of the inhibitors (ZSTK474, Wortmannin, KP372-1 and Rapamycin) used in this study are indicated.

The mTOR kinase acts as an Akt substrate when mTOR binds to Raptor to form mTORC1. But mTOR can become an Akt upstream activator when mTOR binds to Rictor to form mTOR complex 2 (mTORC2) (reviewed in ref. [23]) mTORC1 promotes protein synthesis through activation of its two downstream pathways: p70S6 kinase (p70S6K)/S6 ribosomal protein (S6RP) pathway triggers translation of 5' terminal oligopolypyrimidine (TOP) mRNAs encoding ribosomal proteins and elongation factors and eukaryotic translation initiation factor 4E (eIF4E)-binding protein 1 (4E-BP1)/eIF4E pathway initiates cap-dependent translation [24-30]. Accumulating evidence shows that regulation of eIF4E activity is a two-step mechanism. Initially, active mTORC1/4EBP1 signaling causes dissociation of eIF4E from 4EBP1 binding, which in turn allows Erk and/or p38 MAPK-mediated MnK1 and Mnk2 to phosphorylate eIF4E on ser209, consequently facilitating eIF4E to enter the eIF4F complex and triggering cap-dependent translation [27-30]. The cap-dependent translation can synthesize proteins promoting cell growth (e.g. cyclin D1 and c-Myc) and neovascularization (e.g. VEGF, bFGF) and some malignant behaviours associated with tumour progression (e.g. matrix metalloprotease 9) (Figure 1) (reviewed in ref. [31,32]).

It has been reported that a variety of molecular alterations in any component of the PI3K pathway and its upstream signals can lead to constitutive activation of PI3K kinase cascades. This includes mutations identified in genes encoding RTKs such as mutant KIT-driven human and canine mast cell tumours and mutant Flt3-driven leukemia [33-35]. Mutations of K-ras and N-ras genes have been documented in canine lung cancer and canine leukemia respectively [36]. Aberrant expression of class I PI3K subunits, such as amplification of *PIK3CA* and mutation of *PIK3R1*, is commonly found in colon cancer [37,38]. High frequency of *PTEN* mutation has been reported in malignant glioblastoma [39]. In addition, post-translational modification of PTEN, leading to down-regulation of PTEN activity, has been described in T cell leukemia [40]. Alterations of three Akt isoforms, including amplification of *Akt1*, somatic (activating) mutations of *Akt1*,amplification of *Akt2*, overexpression of *Akt2* without evidence of *Akt2* amplification, overexpression of *Akt3* mRNA and protein but lack evidence of *Akt3* amplification, and somatic (activating) mutations of *Akt3* have been reported in a wide range of tumour types [41-46].

In this study, we examined the importance of the class I PI3K/Akt pathway in promoting tumourigenicity of canine cell lines by utilizing small molecules ZSTK474, KP372-1 and Rapamycin that selectively inhibit class I PI3K, Akt and mTOR, respectively. Canine lines were treated with these inhibitors and cell survival determined by CellTiter-Glo assays and annexin V/PI staining, whilst activation of PI3K/Akt/mTOR components were detected by western blotting. This paper demonstrates that class I PI3K/Akt signaling is critical for the viability of all canine cancer cell lines studied. In particular, Akt-mediated anti-apoptotic activity was found to be critical for maintaining cell viability. Furthermore, we demonstrate that simultaneous inhibition of class I PI3K and mTOR may offer a better therapeutic approach for canine cancer therapy than the concomitant treatment of the PI3K pathway in combination with conventional cancer cytotoxic drugs.

## Results

### Class I PI3K signaling is activated in canine cancer cells

**Figure 2:**
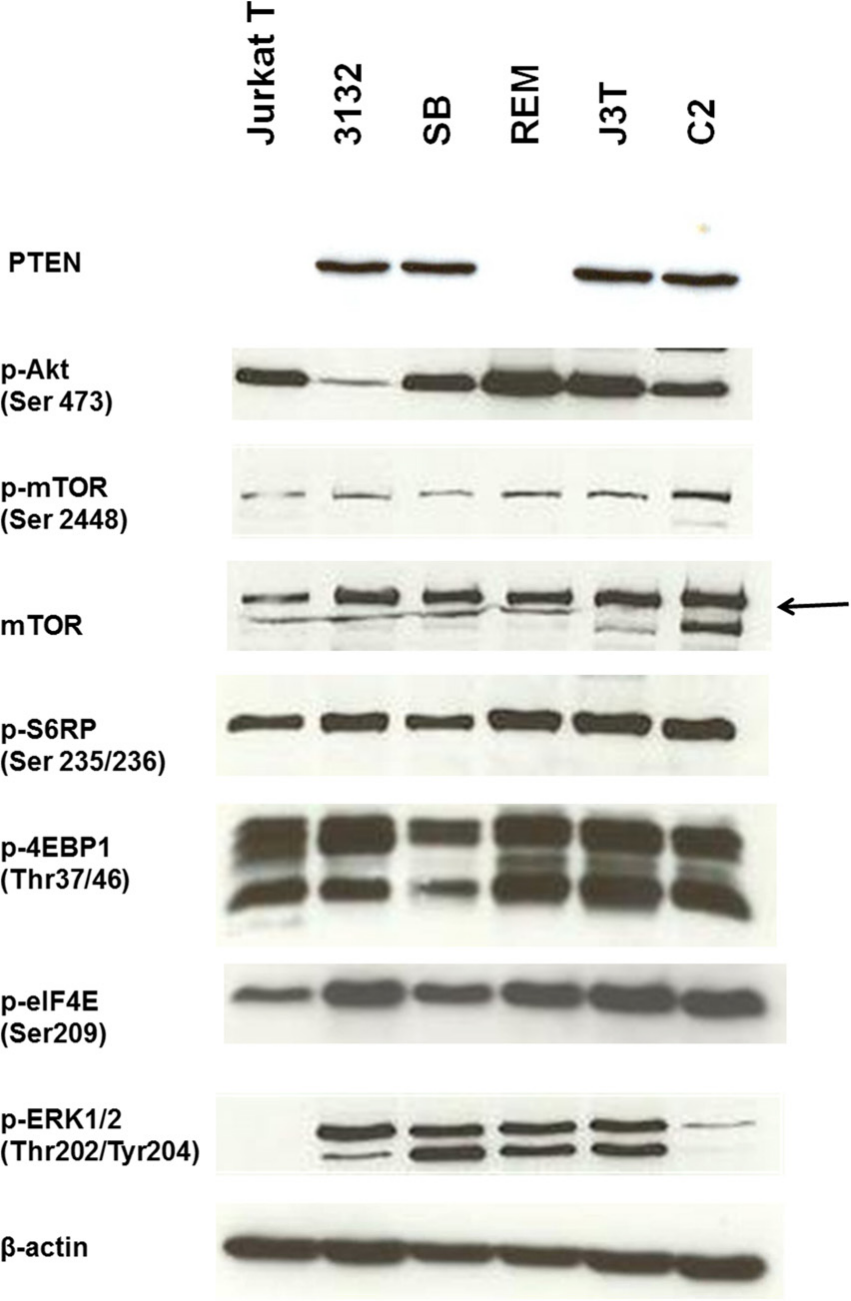
**Western blot analysis of components of the class I PI3K and ERK pathways in human and canine cancer cells.** Whole cell lysates (comprising 50 μg total protein) were subjected to western blotting analysis with β-actin as a loading control.

To determine the extent of class I PI3K kinase pathway activation in these five canine tumour cell lines, we employed western blot analysis to examine the presence of active (phosphorylated) forms of several components of the class I PI3K pathway, including phosphorylated Akt, mTOR, S6RP, 4EBP1 and eIF4E. In addition to these canine cell lines, the human Jurkat T leukemic cell line was used as control as the cell line has constitutive activation of class I PI3K signaling through PTEN loss [47]. As shown in Figure 2, all canine lines with either PTEN expression (3132, SB, J3T and C2 cells) or PTEN loss (REM cells) expressed detectable levels of active forms of these proteins, indicating active class I PI3K signaling in these canine cells.

Because accumulating evidence suggests cross-talk between class I PI3K and Ras/Raf/ERK MAPK pathways commonly occurs (reviewed in ref. [48]), we explored the activity of the ERK/MAPK pathway in these canine cells. Our western blot results demonstrated that these canine cells expressed detectable levels of active forms (phosphorylation) of ERK1/2, indicating Ras/ERK MAPK signaling is also activated in these canine cells. However, this was not detected in the human Jurkat cell line and very low in the canine C2 cell line (Figure 2).

### Inhibition of class I PI3K/Akt/mTOR signaling significantly decreases the viability of canine cancer cell lines

**Figure 3:**
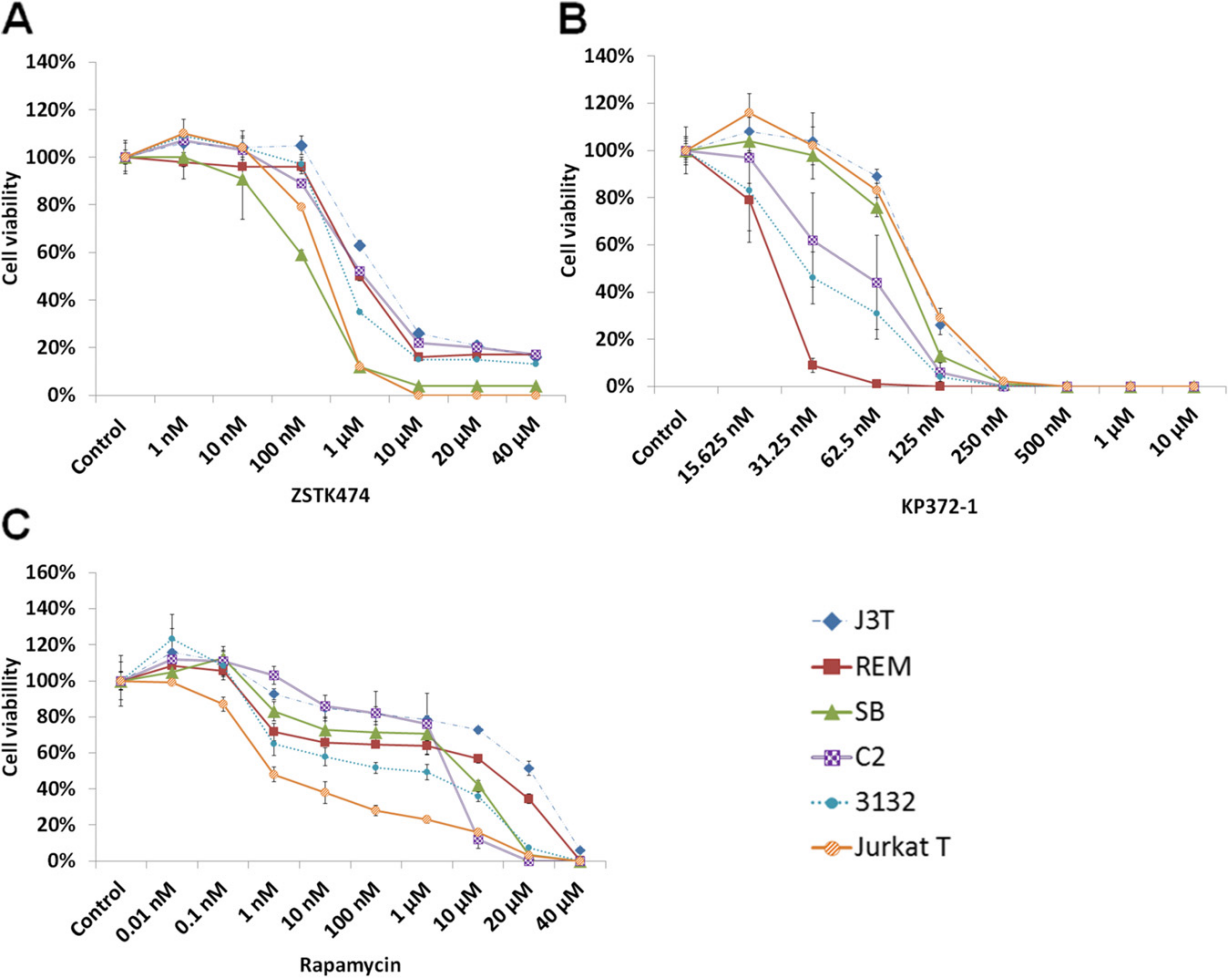
**Sensitivity of canine and human cancer cells to inhibitors targeting class I PI3K/Akt/mTOR pathway.** Cells were treated with a range of doses of the pan-class I PI3K inhibitor ZSTK474 for 3 days (**A**), Akt inhibitor KP372-1 for 2 days (**B**), or mTOR inhibitor Rapamycin for 3 days (**C**). After drug treatment, the number of viable cells was determined by using CellTiter-Glo® Luminescent Cell Viability Assay. Results were expressed as mean (±SD) counts of quadruplicate wells. The values of the viability rates of the drug-treated cells were compared with the vehicle (DMSO)-treated cells on the same culture plates.

To investigate the potential role of class I PI3K signaling in canine cell lines, we used specific chemical inhibitors to block pathway components. Inhibitors used were ZSTK474, KP372-1 and Rapamycin, which targeted pan-class I PI3Ks, Akt and mTOR respectively. Subsequently, we compared cell viability of drug-treated cells with those of vehicle-treated cells by using a standard cell viability assay. While we recognize that colony-forming assays represent a more robust method for measuring responses to anti-cancer agents, this would have been impractical for such a large-scale cell study. As shown in Figure 3A, ZSTK474 at concentrations between 100 nM and 10 μM exhibited a remarkable decline in cell viability by ≥74% with almost full inhibition in SB (96%) and in Jurkat T cells (100%). However, the effect of this drug at concentrations between 10 μM and 40 μM appears to plateau in J3T, C2 and 3132 cells with no further inhibition in REM and SB cells. In this study, KP372-1 showed its efficient inhibition effects on all cell lines causing 100% loss in cell viability after incubation with this compound at the concentrations of ≥ 250 nM for 2 days, compared with ZSTK474 and Rapamycin which required a longer period of time (3 days) and much higher doses (at micromolar concentrations) to reach effective inhibition (Figure 3). Notably, REM cells were most sensitive to KP372-1 with full inhibition of cell viability at the concentration of ≥ 62.5 nM.

With regard to Rapamycin, it was observed that the doses within a nanomolar range had limited effects on inhibiting the viability of these canine cells. Jurkat T cells were observed to be most sensitive to Rapamycin (concentration for 50% inhibition (IC-50) of viability ~ 1nM) whereas all canine cancer cell lines were relatively resistant to Rapamycin and the IC50 values for canine 3132, C2, SB, REM and J3T cells were 1 μM, 1-10 μM, 10 μM, 10-20 μM and > 20 μM, respectively. Among all lines, canine J3T and REM cells were most resistant to Rapamycin. The doses for Rapamycin to reach full inhibition of all lines were between 20 μM and 40 μM (Figure 3C). The concentrations required to inhibit the target via western blot analysis correlated well with those to cause cell killing via the viability assay.

### The class I PI3K/Akt/mTOR inhibitors abrogate activity of class I PI3K signaling

To study the inhibitory effects of ZSTK474, KP372-1 and Rapamycin on the class I PI3K/Akt/mTOR axis signaling in canine cells, we performed western blot analysis to evaluate expression levels of active (phosphorylated) forms of class I PI3K downstream effectors, including Akt, S6RP, 4EBP1 and eIF4E.

**Figure 4:**
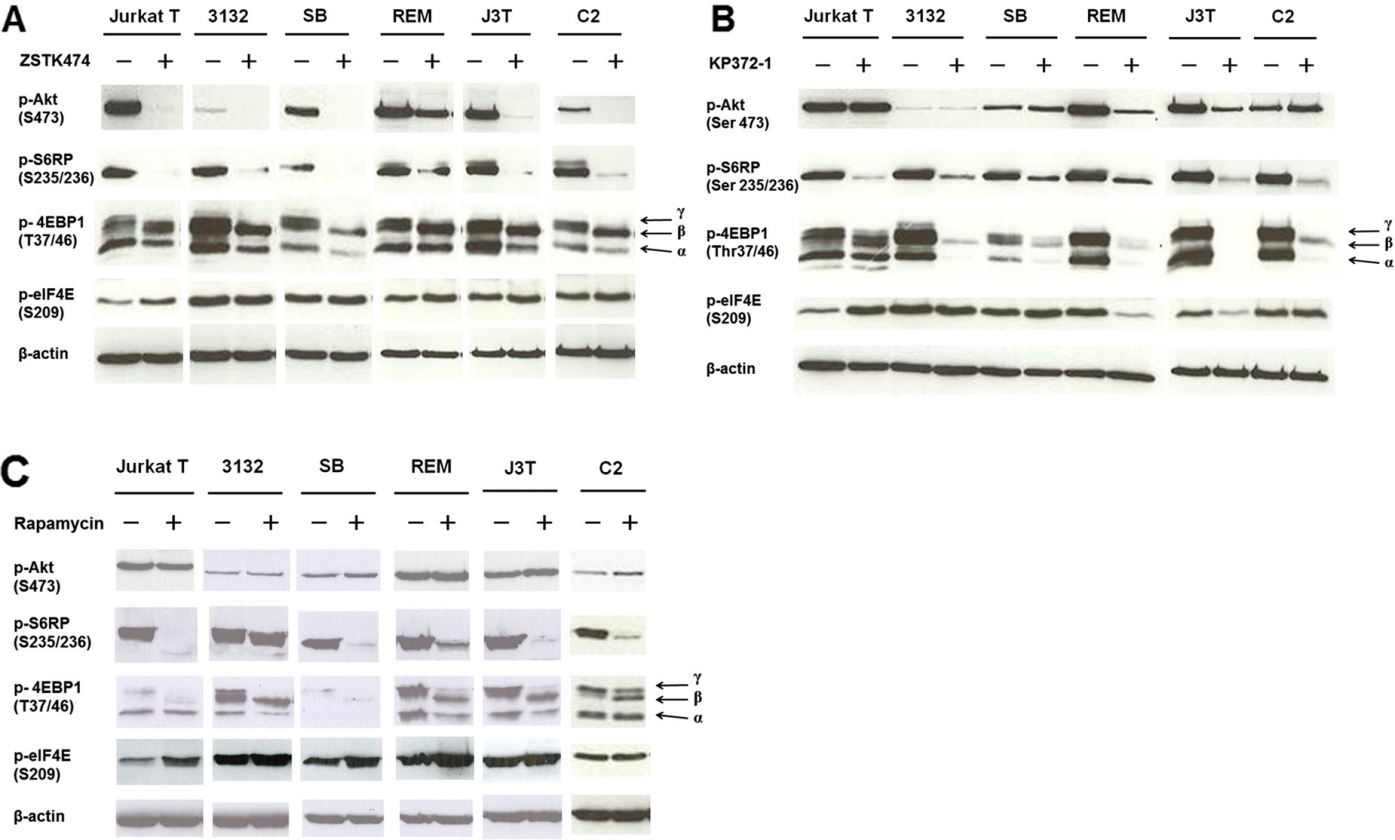
**Effects of the inhibitors on class I PI3K/Akt/mTOR axis signaling in canine and human cancer cells.** Cells were seeded at a density of 20,000 cells per ml overnight, followed by treatment with 1 μM ZSTK474 (**A**), 400 nM KP372-1 (**B**), or 100 nM Rapamycin (**C**) for 5 hrs. Whole cell lysates (comprising 50 μg total protein) were subjected to western blot with the indicated antibodies. β-actin was used as a loading control.

Western blot analysis demonstrated that ZSTK474 down-regulated phosphorylation of Akt and mTOR downstream targets S6RP and 4EBP1. However, there was no change in phosphorylation of eIF4E (Figure 4A). KP372-1, at the concentration of 400 nM, down-regulated phosphorylation levels of S6RP and 4EBP1 in all lines and eIF4E in J3T and REM cells. However, this inhibitor was observed to up-regulate phosphorylation levels of eIF4E in Jurkat T cells (Figure 4B). Rapamycin inhibited mTORC1 signaling, based on decreased γ hyper-phosphorylation of 4EBP1 and phosphorylation of S6RP. But up-regulation of eIF4E phosphorylation was observed in human Jurkat T cells upon Rapamycin treatment (Figure 4C).

**Figure 5:**
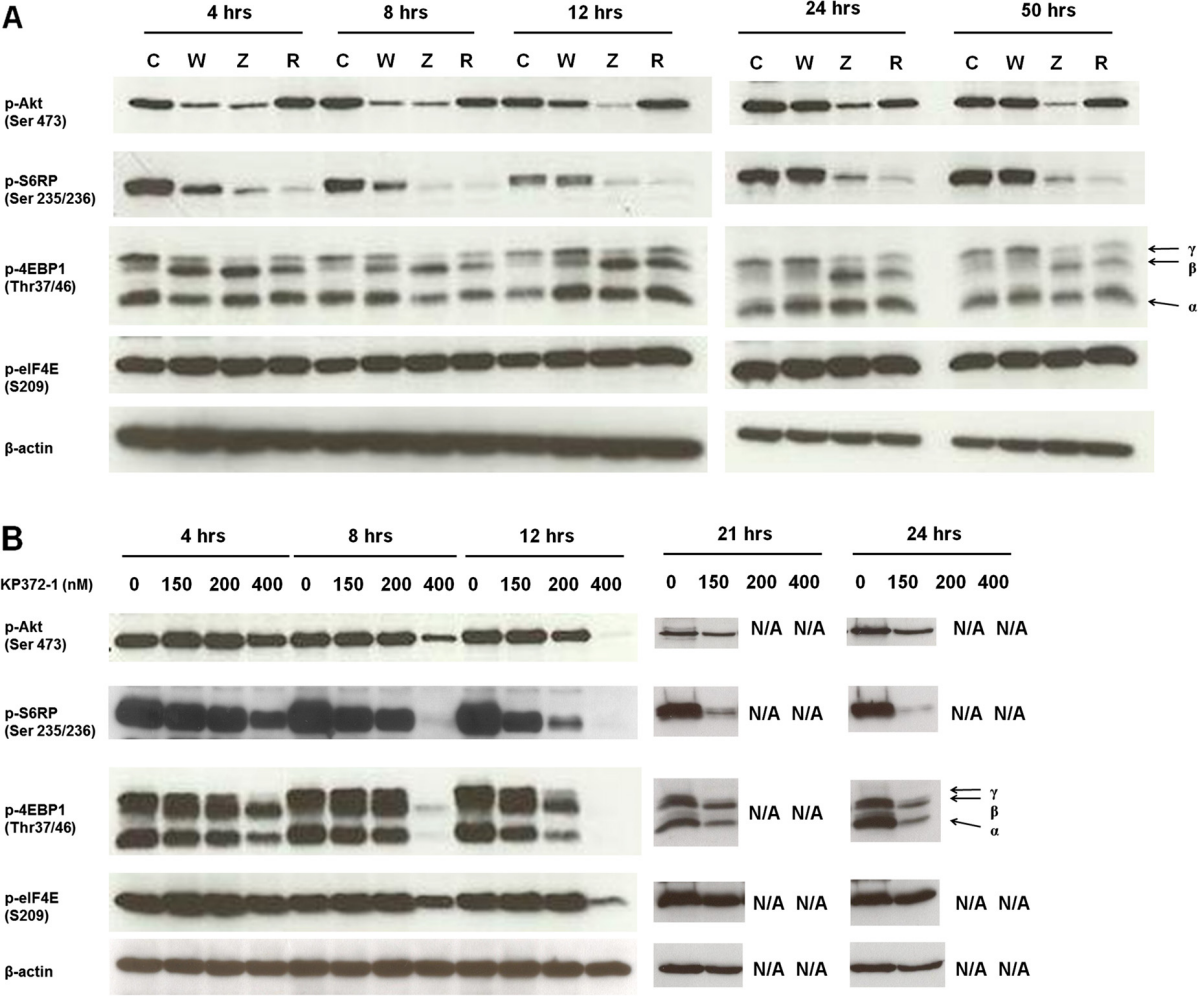
**Effects of the inhibitors on class I PI3K/Akt/mTOR axis signaling in canine C2 cells.** Cells were treated with pan-class I PI3K inhibitor Wortmannin (W) at 1 μM and ZSTK474 (Z) at 1 μM, mTOR inhibitor Rapamycin (R) at 100 nM (**A**) and Akt inhibitor KP372-1 at 0, 150, 200 and 400 nM (**B**) for the indicated period of time. Whole cell lysates (comprising 50 μg total protein) were subjected to western blot with the indicated antibodies. β-actin was used as a loading control. N/A indicates data unavailable due to induction of apoptosis in all cells.

To dissect the dynamics of inhibition further, we performed a time-course study utilizing the C2 cell line only. As shown in Figure 5A, ZSTK474 and Wortmannin, both of which are inhibitors targeting all isoforms of p110 subunits of class I PI3K, blocked class I PI3K activity, as evidenced by significant reduction in phosphorylation levels of Akt and its downstream substrates S6RP and the γ hyper-phosphorylated form of 4EBP1 in C2 cells. However, compared with Wortmannin, ZSTK474 showed greater potency and greater duration of activity in down-regulating class I PI3K kinase signaling. This was based on the results showing that inhibition of phosphorylation of downstream elements of class I PI3K by ZSTK474 lasted for 50 hrs whereas Wortmannin lasted for 12 hrs (Figure 5A). The efficacy of Rapamycin in inhibiting mTORC1 signaling lasted for 50 hrs, as indicated by decreasing phosphorylation levels of S6RP and γ hyper-phosphorylation form of 4EBP1. This is consistent with previous studies suggesting that the efficacy of Rapamycin can last for ~3 days [9].

For the time course study of KP372-1 in C2 cells, three doses higher than the inhibitory concentration of 100% cell viability (IC-100), including 150, 200 and 400 nM, were tested. At the highest dose (400 nM), the phosphorylation levels of PI3K/Akt substrates S6RP and 4EBP1 were decreased at 4 hrs. However, at 8 and 12 hours, this dose demonstrated profound inhibition of phosphorylation of all PI3K downstream substrates, including Akt, S6RP, 4EBP1 and eIF4E, (Figure 5B). KP372-1 at concentrations between 150 nM and 200 nM showed no inhibitory effects on class I PI3K activity at the early time points of 4 and 8 hrs but gradually down-regulated all of its downstream components at later time points of 12, 21 and 24 hrs (Figure 5B). However, data of C2 cells treated with 200 nM and 400 nM KP372-1 at later time points 21 and 24 hrs were unavailable (Figure 5B).

### Effects of class I PI3K/Akt/mTOR inhibitors on cell apoptosis

**Figure 6:**
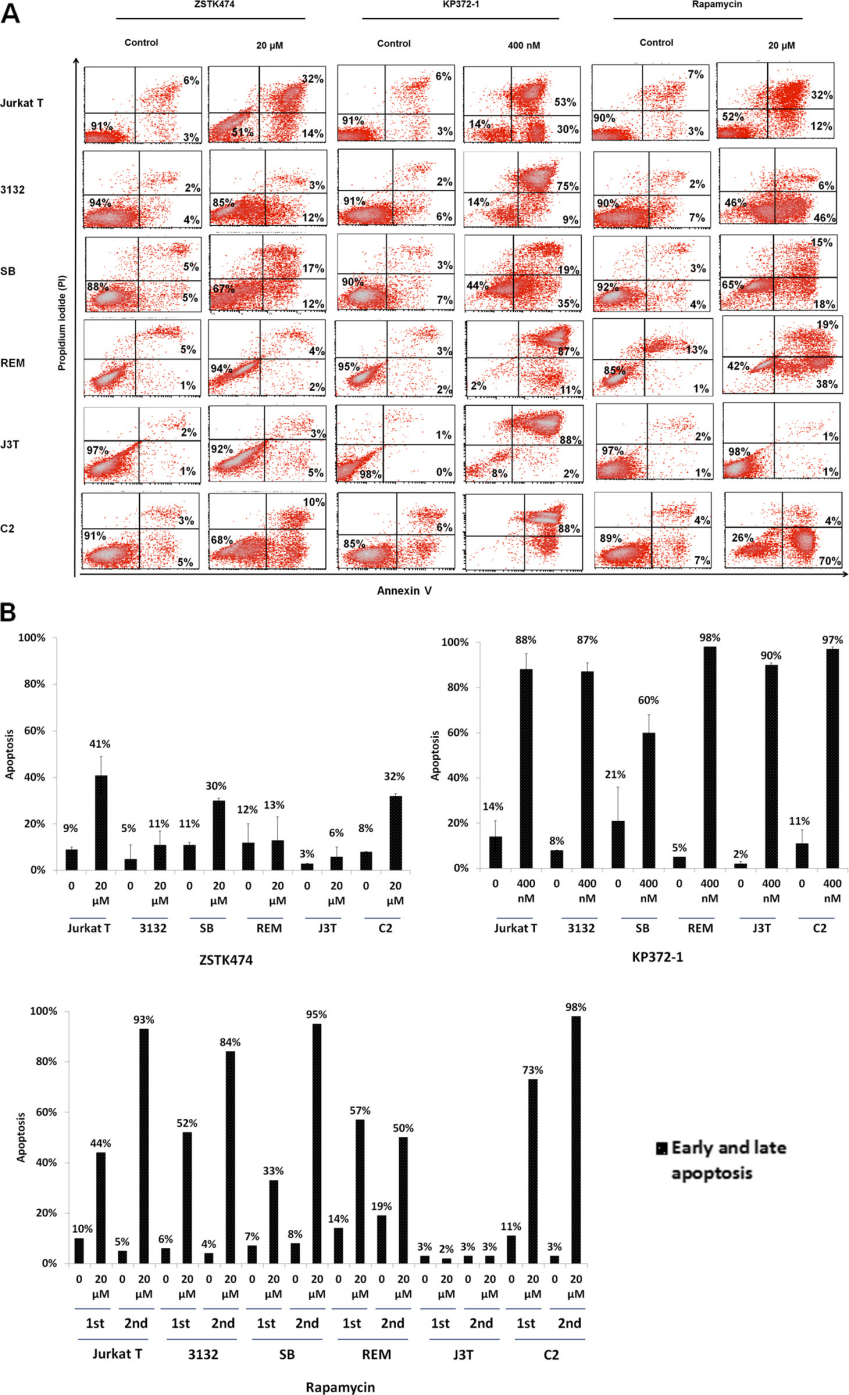
**Effects of ZSTK474, KP372-1 and Rapamycin on induction of apoptosis.** Cells were treated with 20 μM ZSTK474 for 2 days, 400 nM KP372-1 for 1 day, 20 μM Rapamycin for 2 days, or vehicle control as described in Materials and Methods. Induction of apoptosis was determined by annexin V/propidium iodide (PI) staining and flow cytometry analysis. Scatter pots with percentages of live, early apoptosis and late apoptosis were indicated at lower left, lower right and upper right quadrants, respectively in A while B demonstrates this data in a bar chart format. This data is representative of two independent experiments.

To determine whether the three class I PI3K pathway inhibitors ZSTK474, KP372-1 and Rapamycin induce apoptosis in these canine lines, cells were stained with annexin V, a cell apoptosis marker, and propidium iodide (PI), followed by flow cytometry analysis. The results demonstrated that ZSTK474 significantly increased apoptosis of Jurkat T, C2 and SB cells by 32%, 24% and 19%, respectively, as compared with the controls (Figure 6B). Conversely, 3132, J3T and REM cells were not affected by ZSTK474 treatment and the increased apoptosis rate was below 6%. By contrast, KP372-1 was shown to be a potent inducer of apoptosis causing > 87% cell loss in most cell lines and 60% loss of SB cells at the concentration of 400 nM for 1 day. Since Rapamycin at 20 μM was observed to fully inhibit the viability of most cell lines, except REM and J3T cells whose viability rates were reduced by 65% and 48% respectively (Figure 3C), it raised the question whether Rapamycin at such a high dose (20 μM) could down-regulated cell viability through triggering apoptosis. As shown in Figure 6B, apoptotic rates were significantly increased by 20 μM Rapamycin in all lines except J3T cells which was not affected by this drug treatment regime.

### Additive or synergistic inhibitory effects on cell viability when ZSTK474 and Rapamycin were combined

We have demonstrated that Rapamycin inhibited canine cell lines with IC50 values of between 1 and > 20 μM (Figure 3C). Notably, 1 μM is higher than the recommended concentration of Rapamycin or rapalogues that are currently utilized to treat human (Cmax = ~600 nM) and canine cancer patients (Cmax = ~10 nM) due to the drug-related toxicity observed in human patients [49-51]. To investigate whether concurrent inhibition of two other pathway components could improve the efficiency of Rapamycin, cells were concomitantly treated with ZSTK474 and Rapamycin.

**Figure 7:**
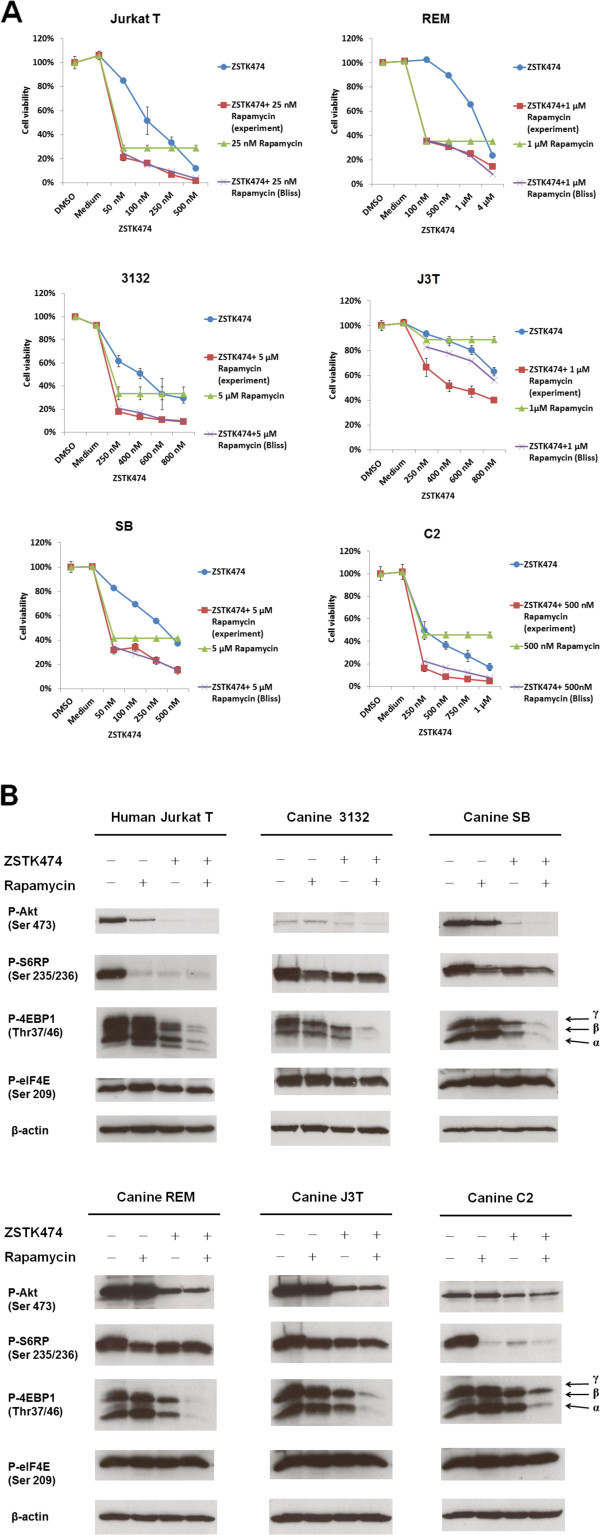
**Effects of ZSTK474 in combination with Rapamycin on cells.** Cells were treated with the indicated doses of ZSTK4747, Rapamycin, the combination of the former two inhibitors or vehicle control for 3 days. Additive, synergistic, or antagonistic inhibitory effects of the drug combination on cell viability were determined when the experiment values (cell viability percentages) of the drug combination were overlapped with, lower than, or higher than Bliss theoretical values respectively (**A**). Western blot analysis was performed to determine the inhibitory effects of ZSTK474 in combination with Rapamycin on class I PI3K activity (**B**). Cells were seeded at a density of 20,000 cells per ml overnight, followed by treatment with 5 μM ZSTK474 (10 μM ZSTK474 in REM cells), 100 nM Rapamycin, the combination of the former two inhibitors or vehicle control for 18 hrs. Whole cell lysates (comprising 50 μg total protein) were subjected to western blot with the indicated antibodies. β-actin was used as a loading control.

The inhibitory effect of drug combinations on cell viability was evaluated using the Bliss additivism model [52]. Briefly, if the cell viability rates generated by Bliss additivism model analysis were higher than, overlapped with, or lower than those rates obtained from experimental results, it was assumed that the combination had a synergistic, additive, or antagonistic effect, respectively. As shown in Figure 7A, the Bliss analyses showed that ZSTK474 combined with Rapamycin had an additive effect on most lines and even a synergistic effect on J3T cells. In this study, this drug combination demonstrated an increased efficacy of: 8-22% in Jurkat, 16-23% in 3132, 7-22% in SB, 0-10% in REM, 23-36% in J3T and 13-29% in C2, as compared with either Rapamycin or ZSTK474 alone, depending on which single agent achieved maximal inhibition of cell viability. Notably, canine J3T cells, as mentioned earlier (see Figure 3C), were most resistant to Rapamycin but showed synergistic response to the drug combination, suggesting that class I PI3K/Akt signaling may be activating a cell survival pathway other than mTOR.

Further, western blot analysis, demonstrated that ZSTK474 alone or in combination with Rapamycin significantly decreased the levels of phospho (p)-Akt in most cell lines but moderately decreased p-Akt in C2 cells (Figure 7B). P-Akt levels in Jurkat T cells were decreased by Rapamycin after incubation for a longer time period (18 hrs). Similar effects of Rapamycin on Jurkat T cells and other cell lines after exposure for 24 hrs, have been described in previous studies [53,54]. It was observed that the drug combination profoundly inhibited the levels of p-4EBP1 but not p-S6RP as compared with each drug alone. However, full inhibition of p-4EBP1 did not contribute to down-regulation of p-eIF4E. In Jurkat T cells, Rapamycin-induced phosphorylation of eIF4E was observed to be repressed by co-treatment of Rapamycin in combination with ZSTK474.

### Effects of the combination of the class I PI3K/Akt/mTOR pathway inhibitors and Doxorubicin on SB and REM cells

**Figure 8:**
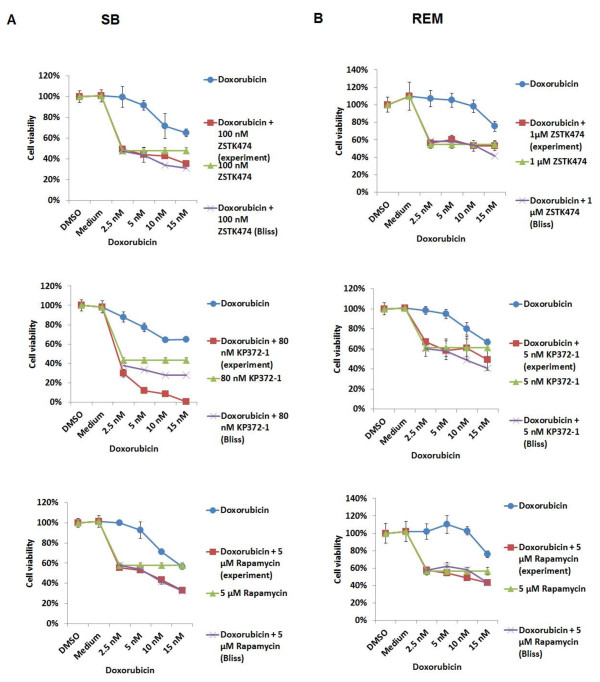
**Effects of the combination of the class I PI3K/Akt/mTOR pathway inhibitors and Doxorubicin on cell viability.** SB (**A**) and REM (**B**) cells were treated with the indicated does of the class I PI3K/Akt/mTOR pathway inhibitors, Doxorubicin, the combination of the former two drugs or vehicle control for 3 days (2 days for KP372-1). Additive, synergistic or antagonistic inhibitory effects of the drug combination on cell viability were determined when the experiment values (cell viability percentages) of the drug combination were overlapped with, lower than, or higher than Bliss theoretical values respectively.

To investigate the impact of inhibition of PI3K/Akt/mTOR axis pathway on the chemosensitivity of canine tumours, we evaluated the effects of the combination of the class I PI3K pathway inhibitors and Doxorubicin on the viability of canine SB and REM cells and utilized the Bliss additivism model to analyze the effects. As shown in Figure 8, the Bliss analysis showed that ZSTK474 antagonized the cytotoxic effects of Doxorubicin in both cell lines. KP372-1 highly synergized with the cytotoxic action of Doxorubicin in SB cells with an increase in efficacy of 13-43%, as compared with treatment with KP372-1 alone. There was antagonism between the actions of KP372-1 with Doxorubicin in REM cells. Rapamycin was observed to enhance Doxorubicin-induced cytotoxicity in both cell lines in an additive manner with an increase in efficacy of 2-23% in SB cells and 2-13% in REM cells as compared with either Rapamycin or Doxorubicin alone.

## Discussion

In the present study, we demonstrate that human and canine cancer cell lines express constitutively activated class I PI3K/Akt/mTORC1 axis signaling, as evidenced by detectable levels of phosphorylated forms of PI3K downstream effectors, including Akt, mTOR, S6RP, 4EBP1 and eIF4E. Subsequently, we inhibited the class I PI3K pathway at different levels by utilizing small molecules inhibitors ZSTK474, KP372-1 or Rapamycin to specifically target pan-class I PI3K, Akt and mTOR respectively. Previous studies have demonstrated ZSTK474 to have ~11, ~24, and ~27 fold specific inhibition for class I PI3Kα over class II PI3K-C2β, mTOR and DNA-dependent protein kinase (DNA-PK), respectively [55,56]. Moreover, this inhibitor is reported to have weak or no inhibitory effects on activities of class II PI3K-C2α, class III PI3K, and PI4K. In addition, ZSTK474 did not down-regulate phosphorylation of ERK and activities of several components of MAPK pathway [55-58]. Therefore, our results suggest that the viability of the cell lines tested is, in part class I PI3K-dependent. However, we also observe that ZSTK474 fails to fully inhibit cell viability in most canine cell lines, suggesting the existence of another mechanism for cell survival. The active ERK signaling detected in these canine cells may play a role in resistance to PI3K pathway inhibition.

Western blot analysis demonstrated that ZSTK474 inhibits the class I PI3K/Akt/mTOR axis signaling. Analysis of apoptosis revealed that ZSTK474 is less potent at apoptosis induction than KP372-1 or Rapamycin, suggesting that ZSTK474 does not inhibit cell viability entirely through induction of apoptosis. A recent study of human cancer cell lines showed that ZSTK474 has potent effects on arrest of cell cycle progression through inhibition of phosphorylation or expression of Akt and/or mTORC1 substrates, such as p-GSK3β, p-mTOR, p-p70S6K and cyclin D1. However, ability to induce apoptosis is cell line dependent and is considered, in general, a weak inducer of apoptosis [56]. Our study suggests that class I PI3K is critical to the viability of cancer cell lines but implicates the mechanism of ZSTK474 to be through inhibition of Akt/mTORC1-mediated protein synthesis and cell growth rather than apoptosis induction.

In this study, KP372-1 is observed to be the most potent drug to down-regulate cell viability, indicating the critical role for Akt in these cell lines. Western blot analysis demonstrated that high doses or long drug exposure of KP372-1 is required to inhibit Akt/mTORC1 signaling compared to ZSTK474 and Rapamycin. However, KP372-1 showed remarkable efficacy for inducing apoptosis. A previous study of KP372-1 on acute myelognous leukemia (AML) suggests that this drug predominantly acts on inhibition of PDK1/Akt-mediated anti-apoptosis mechanism but has no function on arresting cell cycle progression [59]. In agreement with this study, our data suggests that KP372-1 is a potent inducer of apoptosis through down-regulation of Akt-mediated survival mechanism but has less effect on inhibition of Akt/mTORC1-mediated activities such as protein synthesis and cell cycle progression. In addition, as REM cells are highly sensitive to KP372-1 but relatively resistant to Rapamycin, it is suggested that Akt-mediated anti-apoptosis activity, not mTORC1 activity, is critical for the viability of REM cells. In the time course study of C2 cells, we find that KP372-1 at 400 nM initially down-regulates phosphorylation of mTORC1 substrates S6RP and 4EBP1, and then gradually down-regulates phosphorylation of Akt and eIF4E. We show that 400 nM KP372-1 induces most C2 cells to apoptosis after 24 hours of incubation, indicating the correlation of protein loss with apoptosis. The down-regulated phosphorylation of Akt and eIF4E may be a late event of de-phosphorylation of all protein kinases when most cells undergo apoptosis. In addition to C2 cells, decreased phosphorylation of all class I PI3K substrates is also observed in KP372-1 treated REM and J3T cells.

The effects of Rapamycin on the viability of canine cells tested in this study and the apoptosis results are in agreement with previous findings that higher doses (micromolar ranges) of CCI-779 or Rapamycin can overcome drug resistance mechanism and achieve full inhibition of cell proliferation through the inhibition of mTORC2-mediated Akt and ERK survival pathways and the profound inhibition of global protein synthesis [60,61]. Accumulating evidence suggest that Rapamycin at lower doses (nanomolar range) requires initial interaction with cytoplasmic receptor FKBP12, which in turn allows Rapamycin to bind mTORC1, leading to inhibition of mTORC1 pathway but also generation of drug resistance [60,61]. So far, at least three mechanisms have been reported to be associated with Rapamycin-resistance and all of them are linked to mTORC1 inhibition. First route is through inhibition of mTORC1/p70S6K, which in turn releases the feedback loop of p70S6K/IRS-1/PI3K/Ras and stimulates Ras/ERK MAPK and PI3K/Akt pathways [62-64]. The second route is through inhibition of mTORC1, which in turn activates expression of insulin-like growth factor-1 (IGF-1) and IRS-2, followed by activation of IGF-1/IGF-1 RTK/IRS-2/PI3K with a consequence of activation of the PI3K/Akt pathway [65]. The third route is through mTORC1 inhibition, followed by activation of the c-SRC/RTK pathway and subsequent activation of the Ras/ERK MAPK pathway [66]. Our western blot data show that low doses (100 nM) of Rapamycin inhibits mTORC1 signaling but stimulates phosphorylation of eIF4E in Jurkat T cells. As eIF4E phosphorylation is under the control of ERK and/or p38 MAPK pathways following mTORC1-mediated dissociation from 4EBP1, it is suggested that Rapamycin at the low dose stimulates ERK or p38MAPK/Mnk/eIF4E pathway in Jurkat T cells through any of the three Rapamycin-resistance mechanisms described above [27-30]. Indeed, a previous study of a PIM inhibitor has demonstrated that inhibition of p70S6K activity in Jurkat T cells triggers a p70S6K/IRS-1 feedback loop and activates Ras/MAPK signaling [67]. In this study, we find that both Rapamycin and KP372-1 significantly increase phosphorylation of eIF4E in this cell line and the Rapamycin-induced phosphorylation of eIF4E in Jurkat T cells is suppressed by Rapamycin in combination with ZSTK474. Another study has reported that Rapamycin-induced eIF4E phosphorylation can be reversed by the combination of Rapamycin and a PI3K inhibitor but, in certain cell lines, PI3K inhibitor alone can still increases eIF4E phosphorylation [63]. This suggests that tumour cells can escape cell death through additional mechanisms other than the p70S6K/IRS-1/PI3K/Ras feedback loop. Due to simultaneous inhibition of both class I PI3K and mTORC1 reversing Rapamycin-induced eIF4E hyper-phosphorylation, it is suggested that Jurkat T cells are resistant to Rapamycin through either activating the p70S6K/IRS-1/PI3K/Ras or IGF-1/IGF-1 RTK/IRS-2/PI3K pathways, but not through the third resistant mechanism that is the c-SRC/RTK pathway [62,65,66].

By contrast, Rapamycin at higher doses (micromolar range) directly binds to mTOR, which in turn inhibits mTORC2 and global translation processes, leading to a dramatic decline in cell viability [61]. A recent study shows that inhibition of mTORC2 by silencing expression of the Rictor subunit can not only down-regulate Akt signaling but can also down-regulate ERK phosphorylation [60]. In this study, we have shown that Rapamycin at a high dose such as 20 μM significantly increases apoptotic rates of most cell lines, confirming that reduction of cell viability was in part through apoptosis. Hence, our data support previous findings that high doses of Rapamycin decrease global translation processes and down-regulate mTORC2 activity [61]. Notably, mTORC2 has recently been identified as activators of not only Akt survival kinase but also serum- and glucocorticoid-induced protein kinase (SGK), a pro-survival factor, and protein kinase C (PKC) [68-70]. This implicates a role of mTORC2 in promoting survival of these canine cancer cell lines tested in the present study.

It is suggested that the mechanism for the additive or synergistic effects of ZSTK474 and Rapamycin on cells is through simultaneous inhibition of Akt activity and inhibition of mTORC1 activity. However, this drug combination has no effects on eIF4E phosphorylation, in agreement with previous findings that eIF4E phosphorylation is regulated by ERK or/and p38MAPK pathways. Interestingly, we observed that this drug combination does not profoundly inhibit phosphorylation of S6RP in most canine cells except C2 cells. As S6RP has been reported to have three upstream activators, which are PDK1/p70S6K, mTORC1/p70S6K and Ras/ERK/RSK pathways, it is suggested that Ras/ERK/RSK is most likely to contribute to the maintenance of S6RP phosphorylation after blockade of both PI3K and mTORC1 signaling in these four canine cell lines [71-73]. Because simultaneous inhibition of class I PI3K and mTOR by the drug combination can result in down-regulation of PDK1- and mTOR-mediated phosphorylation of PDK1, it is possible that active ERK signaling which is detected in these canine cell lines may support S6RP activity and thus provide an explanation for the limited effects of Rapamycin in the down-regulation of S6RP phosphorylation in some lines such as 3132. In Jurkat T cells, chronic exposure to Rapamycin down-regulates both mTORC1 signaling and Akt phosphorylation, which may provide an explanation for the high sensitivity of Jurkat T cells to Rapamycin. Taken together, the additive/synergistic effects of ZSTK474 combined with Rapamycin suggest the resistance of these canine cells to Rapamycin alone, is due to active Akt and ERK survival pathways.

In summary, our data demonstrates that the class I PI3K/Akt/mTOR pathway is a major signaling axis in the survival of cancer cells. We show that ZSTK474 and KP372-1 effectively down-regulate cell viability, and highlight the critical role of Akt activity in promoting the proliferation and survival of cells. Further, we show that ZSTK474 and KP372-1 inhibit cell viability via different mechanisms. ZSTK474 effectively down-regulates mTORC1 signaling but has weak potency in apoptosis induction. KP372-1 has remarkable efficacy for apoptosis induction but has weak potency on mTORC1 inhibition. Rapamycin at nanomolar concentrations has cytostatic effects. In contrast, Rapamycin at micromolar doses shows cytotoxic effects, suggesting mTORC2 inhibition effectively inhibits the viability of canine cancer cells. We also show that ZSTK474 can enhance the effects of Rapamycin on reducing cell viability, by inhibition of Akt pathways. However, despite the additive or synergistic effects, the overlapping toxicities of these drugs would need to be resolved in a clinical setting. Our data suggest that the effect of combining inhibition of the PI3K/AKT pathway with conventional drugs such as doxorubicin is cell line dependent. However, dissecting this synergistic mechanism may offer an opportunity to identify cancer patients where this approach may be beneficial.

## Conclusion

In conclusion, the results of the present study support the development of canine cancer therapy specifically targeting class I PI3K/Akt pathway. This study also implicates mTORC2 as a potential target for canine cancer treatment. As such mTORC2 deserves further investigation to clarify the correlation of its downstream targets with tumour survival mechanism. In addition, the current data implicate the Ras/Raf/MEK/ERK pathway in resistance mechanisms to class I PI3K pathway inhibitors, supporting recent studies which generally recommend the use of combinatorial inhibitors targeting both PI3K/Akt signaling and Ras/ERK signaling [74,75].

## Methods

### Cell lines and tissue culture

Jurkat T (human T lymphoblast-like cells), 293 T (human embryonic kidney 293 cells containing the SV40 Large T-antigen), 3132 (canine lymphoma of B-cell origin), REM (canine mammary carcinoma), SB (canine hemangiosarcoma), J3T (canine glioma) and C2 (canine mast cell tumour) cells, were used in this study. The Jurkat T, 3132, REM and J3T cells were grown in RPMI 1640 (11835, Gibco, Invitrogen, Paisley, UK), RPMI 1640 (11835, Gibco, Invitrogen, Paisley, UK), DMEM (41965, Gibco, Invitrogen, Paisley, UK) and DMEM (41966, Gibco, Invitrogen, Paisley, UK) media respectively, all of which contained 10% (v/v) fetal bovine serum (FBS), 100 U/ml penicillin and 100 μg/ml streptomycin. The C2 cell line, provided by Dr. Richard Elders, The Royal Veterinary College, London, was grown in Minimum Essential Medium Eagle (M5650, Sigma-Aldrich, Ayrshire, UK) medium containing 5% FBS, 1% non-essential amino acid mix (NEAA) (Sigma-Aldrich, St Louis, MO, USA), 1% GlutaMAX-1 (Invitrogen, Paisley, UK), 50 μg/ml gentamicin. The SB cell line was grown in EBM-2 (CC-3135, Lonza, Walkersville, MD, USA) supplemented with 2% FBS and EGM-2 SingleQuots (CC-4176, Lonza, Walkersville, MD, USA) kit containing 0.04% hydrocortisone, 0.4% hFGF, 0.1% VEGF, 0.1% R3-IGF-1, 0.1% ascorbic acid, 0.1% hEGF, 0.1% GA-1000 and 0.1% heparin.

### Drug compounds and pathway inhibitors

ZSTK474 (pan-PI3K inhibitor, Z-1066, LC Laboratories, USA), Wortmannin (pan-PI3K inhibitor, 1232, Tocris bioscience, USA), KP372-1 (Akt inhibitor, B-0102, Echelon, USA) and Rapamycin (mTOR inhibitor, R0395, Sigma-Aldrich, USA) were dissolved in dimethyl sulfoxide (DMSO) as concentrated stocks that were stored at -70 °C and diluted freshly in cell medium before use. Doxorubicin was purchased from Pharmacia, Pfizer Service Company (Zaventem, Belgium) and was soluble in water.

### Cell viability assay

Cells were seeded at a density of 3 × 10^3^ cells per well in 96-well plates overnight at 37 °C with 5% CO_2_, followed by incubated with various doses of either single agent or in combination with other drugs, or DMSO vehicle for a period of time. All experiments were performed in at least three replicates. After the drug treatment, the number of viable cells was determined by using CellTiter-Glo® Luminescent Cell Viability Assay (Promega, Madison, WI, USA) according to the manufacturer’s instructions. This commercial kit quantified cell viability by measuring the amount of ATP released from viable cells. The more viable cells were present, the more ATP released and the higher the value of luminescence detected.

### Analysis of apoptosis and cell death

Cells were plated at a density of 3 × 10^4^ cells per ml and incubated overnight at 37 °C with 5% CO_2_. After that, cells exposed to treat with 20 μM ZSTK474 for 2 days, 400 nM KP372-1 for 1 day, 20 μM Rapamycin for 2 days or vehicle control were collected for apoptosis analysis by using FITC Annexin V Apoptosis Detection Kit I (556547, BD Pharmingen™, San Diego, CA, USA). In brief, harvested cells were washed with cold PBS and re-suspended in 100 μl of 1x Binding Buffer, followed by stained with FITC Annexin V antibody and propidium iodide (PI) for 15 min in the dark at room temperature, according to the manufacturer’s instructions. Cells were analyzed by flow cytometry using FACS Calibur Flow Cytometer and CellQuest software (BD Biosciences, San Jose, California).

### Preparation of cell lysates and western blotting

Cells were seeded at a density of 20,000 cells per ml overnight at 37 °C with 5% CO_2_, followed by incubated with various doses of either single agent or in combination with other drugs, or DMSO vehicle for a period of time. After the drug treatment, cells were harvested and washed in cold PBS, followed by lysed in 1% NP40 buffer containing 150 mM KCl, 25 mM Hepes (pH 7.4), 5 mM DTT, 50 mM NaF, and 1 x Complete Mini Protease Inhibitor Cocktail Tablet (Roche, Mannheim, Germany). The protein extracts were quantified by using Quick Start Bradford Protein Assay (Biorad Laboratories, CA, USA) according to the manufacturer’s instruction. 50 μg protein specimens were subjected to the SDS-PAGE, followed by transferred onto nitrocellulose membranes. The membranes were immunoblotted with primary antibodies specific for PTEN, phosphor (p)-Akt (Ser473), mTOR, p-mTOR (Ser2448), p-S6RP (Ser235/236) and p-4E-BP1 (Thr37/46), all of which were purchased from Cell Signaling Technology (Danvers, MA, USA) and were diluted 1:1000 in blocking buffer which was made up of 1X phosphate buffered saline (PBS) solution containing 5% skimmed milk and 0.1% Tween-20 and p-eIF4E (Ser209) and β-actin, both of which were purchased from Abcam (Cambridge, UK) and were diluted 1:5000 and 1:3000 respectively in blocking buffer. Subsequently, the immunoblots were probed with either swine anti-rabbit horseradish peroxidase (HRP) conjugated secondary antibody (1:1000) or rabbit anti-mouse HRP conjugated secondary antibody (1:2000 for detection of β-actin), both of which were purchased from DAKO (Glostrup, Denmark) The blots were developed using Amersham ECL Western Blotting Detection Reagents (GE Healthcare, Buckinghamshire, UK) and protein bands were visualized on autoradiography film Hyperfilm (GE Healthcare, Buckinghamshire, UK). All antibodies have previously been validated for canine proteins [51].

### Analysis of drug combination effect

The inhibitory effect of two drug combination on cell viability was defined as additivity, synergy and antagony by using Bliss additivism model. The methods of Bliss analysis was adopted from Buck E, et al. [52] Hypothetical curve was generated by using the equation E_bliss_ = E_A_ + E_B_ – (E_A_ x E_B_). While E_A_ represented the percentage of decreased cell viability by drug A, E_B_ represented the percentage of decreased cell viability by drug B. Therefore, if the cell decreased viability (%) of the combination of the two drugs experimentally was greater than E_bliss_, the effect of the combination was considered to be synergistic. On the contrary, if the percentage of decreased viability obtained by an experiment was less than E_bliss_, the effect of the combination would be considered to be antagonistic. In the present study, the Bliss additivity curves were generated by the combination of various doses of drug A and a constant dose of drug B.

### Statistical analysis

For cell viability assays, the values obtained from cell viability assay, as shown in the figures, were compared with the vehicle control on the same culture plates, followed by expressed as percentages of mean values with standard deviations of at least three replicates.

## Competing interests

The authors state that there is no conflict of interest with regards this study.

## Authors’ contributions

YTC was the PhD student who conducted the study. LYP and KALT provided direct laboratory support, contributed to design and final manuscript. DJA designed the study, obtained the funding and was direct supervisor. The manuscript was prepared by YTC and corrected by all other authors. All authors read and approved the final manuscript.
